# Implementation of a mass canine rabies vaccination campaign in both rural and urban regions in southern Malawi

**DOI:** 10.1371/journal.pntd.0008004

**Published:** 2020-01-23

**Authors:** Carlos Sánchez-Soriano, Andrew D. Gibson, Luke Gamble, Jordana L. Burdon Bailey, Dagmar Mayer, Frederic Lohr, Patrick Chikungwa, Julius Chulu, Ian G. Handel, Barend M. deC. Bronsvoort, Richard J. Mellanby, Stella Mazeri

**Affiliations:** 1 The Royal (Dick) School of Veterinary Studies, The University of Edinburgh, Easter Bush Veterinary Centre, Roslin, Midlothian, United Kingdom; 2 The Roslin Institute, Division of Genetics and Genomics, Easter Bush Veterinary Centre, Roslin, Midlothian, United Kingdom; 3 Mission Rabies, Cranborne, Dorset, United Kingdom; 4 Department of Animal Health and Livestock Development, Lilongwe, Malawi; Universidad Nacional Mayor de San Marcos, PERU

## Abstract

Rabies is a devastating zoonotic disease causing nearly 60,000 deaths globally each year. The disease causes Malawi an economic loss of 13 million USD and kills almost 500 people annually. Domestic dogs are the main reservoir for rabies and vaccinating over 70% of the dog population is the most efficient method to reduce its incidence in both humans and canines. However, achieving such coverages is often difficult and depend on many geospatial factors. Rural and pastoral regions are considered difficult to vaccinate efficiently due to low dog densities, and reports of campaigns spanning large areas containing vastly different communities are lacking. This study describes a mass canine vaccination campaign covering rural and urban regions in southern Malawi. The campaign achieved an average vaccination coverage of 83.4% across 3 districts, and vaccinated over 89,000 dogs through a combined static point and door-to-door effort. A dog population of 107,574 dogs was estimated (dog:human ratio of 1:23). The canine population was found to be almost completely owned (99.2%) and mostly kept for security purposes (82.7%). The dogs were mainly adults, males, and not neutered. Regression analysis identified education level and proportion of young dogs as the only factors influencing (positively and negatively, respectively) whether vaccination coverage over 70% was achieved in a region, independently of variables such as population density or poverty. A second regression analysis was performed predicting absolute vaccination coverage. While education level and the proportion of confined dogs were associated with positive vaccination coverage, higher proportions of young animals and female dogs were associated with a decrease in coverage. This study confirms the feasibility of homogeneously vaccinating over 70% of the dogs in a large area including rural and urban communities. These findings can inform the logistics of future campaigns and might be used as a template to facilitate high-number, high-coverage vaccination campaigns to other regions in sub-Saharan Africa.

## Author summary

Since 99% of human rabies cases are caused by dog bites, mass dog vaccination campaigns are to be the most efficient method to eradicate the disease. The annual vaccination of over 70% of the population is needed to stop transmission of rabies and reduce the incidence of the disease in human and dog populations. Despite international efforts, rabies continues to be prevalent in sub-Saharan Africa, where dog vaccination coverages remain suboptimal. Different communities require different approaches depending on socio-economic and spatial factors. This study describes a mass dog vaccination campaign which achieved homogeneous vaccination coverages of over 70% in 3 districts in southern Malawi, including rural and urban regions, and vaccinated over 89,000 dogs. The dog demographics were studied and compared with reports from other regions of sub-Saharan Africa. Logistic regression models were developed to identify the effect of different factors on coverage. While the influence of several variables on absolute vaccination coverage was determined, only education level and the proportion of young animals were shown to have a significant influence on a region achieving adequate coverage. This study demonstrates the feasibility of vaccinating large number of dogs at high coverage across a wide range of geographical areas in southern Malawi.

## Introduction

Nearly 59,000 lives are lost every year due to rabies [1], a disease that is still prevalent and underreported in the majority of developing countries, causing annual losses of 3.7 million disability-adjusted life years (DALYs) and 8.6 billion USD [1]. With a case fatality of nearly 100% once the clinical symptoms appear [2], the effect of the disease is most severe in those regions with limited healthcare facilities and poor availability of post-exposure prophylaxis (PEP), a common situation in many countries in sub-Saharan Africa (SSA) [3]. Rabies in SSA continues to be a major public health issue seldom given the priority needed for its control or eradication [4].

Rabid dogs are responsible for 99% of all cases of human rabies [5], representing the main reservoir for the disease. For this reason, mass canine vaccination campaigns have been demonstrated to be the most effective strategy for the reduction of rabies burden in dog and human populations [6]. Annual immunization of 70% of the dog population is recommended by the World Health Organization (WHO) as the minimum vaccination coverage to be achieved in order to break the cycle of disease transmission and eventually achieve the eradication of rabies [5, 7]. Many pilot rabies vaccination campaigns have been performed across SSA [8, 9], but there have been few attempts at organizing large scale operations covering regions with different housing densities. Despite the fact that dog populations in Africa are considered mostly owned [9], reaching over 70% of the canine population is challenging and highly dependent on local socioeconomic and cultural factors, especially in rural areas with low dog densities. Information regarding dog ecology in most of SSA is lacking, which hinders the development of vaccination campaigns tailored to the needs of each setting. Furthermore, our understanding of factors influencing whether a dog vaccination campaign successfully achieves an adequate coverage is limited. Such knowledge is necessary to identify which aspects of the campaign need to be improved upon in order to increase their effectiveness.

Rabies places a huge burden on Malawi’s economic development with a loss of 13 million USD every year and killing 3 in 100,000 inhabitants annually [1]. We have previously reported an intensive vaccination campaign in Blantyre city, Malawi’s second largest city, vaccinating over 35,000 dogs in 20 days [10, 11]. The campaign consisted of a combined static point (SP) and door-to-door (D2D) effort which achieved a mean vaccination coverage of 79.3%. Data collected during the Blantyre city campaign allowed to identify barriers to attendance to SP clinics, providing guidelines on ways to increase the efficiency of urban SP campaigns [12]. In 2016, the campaign was expanded to the neighbouring Zomba and Chiradzulu districts vaccinating nearly 90,000 dogs [10]. Alongside vaccination, rabies educational campaigns were conducted in schools in the three districts, which has been shown to effectively increase the knowledge of rabies in primary school children [13].

This publication describes a mass dog vaccination campaign performed in 2017 addressing both rural and urban regions, reporting the number of vaccinated dogs and vaccination coverage achieved in the different districts, and characterising the dog population of the region. In addition, factors influencing absolute vaccination coverage and whether adequate coverage (over 70%) was achieved were also identified using multivariable logistic regression models. The results are expected to facilitate the rollout of efficient large-scale dog vaccination campaigns in different geographical settings in SSA.

## Methods

### Ethics statement

The study was approved by the University of Edinburgh Human and Veterinarian Ethics Committees. Verbal informed consent was obtained from every owner before the vaccination of their dog. Free-roaming dogs whose owner could not be identified were vaccinated in accordance with the Government Public Health protocol, since the vaccination campaign was part of a non-research public health operation.

### Study area

The working area for the campaign covered three adjacent districts within southern Malawi. The economy in these districts is mostly agrarian, with the rural regions being divided in small landholdings. Blantyre district has a human population of 1,251,484 inhabitants, of which nearly 64% live in the urban area [3]. Zomba district has 851,737 inhabitants, around 12% of which live in the urban area [3]. Chiradzulu is a mainly rural district with a population of 356,875 inhabitants [3]. Blantyre city and Zomba city are the second and fourth biggest cities in the country, respectively. The dog population in Blantyre city was estimated to be 44,261 dogs (dog:human ratio of 1:18.1) [11], with a population of 73,419 dogs in the whole Blantyre district [14]. There are no current estimations of the dog populations in Zomba and Chiradzulu districts.

### Mission Rabies 2017 vaccination campaign

Mission Rabies [10] has performed annual dog mass vaccination campaigns in southern Malawi since 2015, a campaign which initially focussed on Blantyre city [11]. The 2017 campaign constituted a large-scale operation performed in Blantyre, Zomba and Chiradzulu districts. The working area was divided into 5 regions, according to the level 2 administrative divisions of the country: Urban Blantyre, covering the city of Blantyre; rural Blantyre, covering the rest of Blantyre district; Urban Zomba, covering the city of Zomba; rural Zomba, covering the rest of Zomba district; and (rural) Chiradzulu, covering the area of Chiradzulu district. The districts are subdivided into administrative zones named Extension Planning Areas (EPA). The campaign was advertised via posters, newspapers and educative visits to schools. The SP stage ran from April 22^nd^ to December 13^th^. Working timespans for each region are described in Table 1. In the urban regions, static vaccination points were set up in fixed locations, while for rural regions they were set up in predefined points or *ad hoc* depending on the location and the type of community, with smaller communities being roamed by vehicle, from where the vaccinations were announced. Animals brought to these clinics received parenteral vaccination (Nobivac Rabies, MSD Animal Health), were marked to prevent re-vaccination, and their owners were given a vaccination certificate. The D2D stage, developed to complement the SP vaccinations, ran from April 24^th^ to December 13^th^. An increased effort was put into the D2D vaccination of rural regions in order to bring high coverages across the entire working area. During this stage, the vaccination teams covered different areas of the regions each day, knocking on household doors along their path, and offering vaccination to both indoor dogs and to dogs on the street. Free-roaming dogs with no identifiable owner were also vaccinated, using catching nets for those difficult to approach. All staff involved were trained to perform the vaccination humanely and causing minimum distress to the dog. After each cycle of SP and D2D vaccination, a survey was conducted in order to assess the vaccination coverage achieved. This stage was performed from June 2^nd^ 2017 to March 13^th^ 2018. The area of the three districts was split into 623 working zones: 364 in Blantyre, 315 in Zomba, and 107 in Chiradzulu. In order to carry out the post-vaccination survey, 194 working zones were randomly sampled, obtaining 99 in Blantyre, 57 in Zomba, and 38 in Chiradzulu. The teams covered those 194 working zones carrying out household questionnaires about dog vaccination and gathering information regarding the dog or the owners.

**Table 1:** Timespans for the different stages of the campaign at each region. Post-vaccination surveys for Zomba district were carried out intermittently between the rural and urban regions.

Region	Static Point	Door-to-Door	Post-vaccination survey
**Urban Blantyre**	April 22 –May 23, 2017	April 24 –May 31, 2017	June 2 –June 21, 2017
**Urban Zomba**	June 10 –June 21, 2017	June 12 –June 21, 2017	Aug. 1 –Aug. 11, 2017
**Chiradzulu**	July 3 –Oct. 13, 2017	July 3 –Oct. 13, 2017	Jan. 16 –Feb. 9, 2018
**Rural Blantyre**	July 3 –Dec. 13, 2017	June 28 –Dec. 13, 2017	Feb. 12 –March 13, 2018
**Rural Zomba**	July 11 –Sept. 29, 2017	July 11 –Sept. 29, 2017	July 31 –Oct. 10, 2017

Data collection during the campaign was performed through the Mission Rabies App [15], a web-based platform for smartphones created for the uncomplicated entry and management of field data, creating automatic timestamps for each dog vaccinated along with recording the geographical coordinates. The app facilitated the collection of relevant data on vaccinated dogs such as age, sex and neuter status, as well as additional information regarding the owner or the household. The app also included a path-tracking tool to allow the teams to check the spatial coverage of the districts in real time.

### Data sources

The analysis performed during this study used the data collected from the SP, D2D and post-vaccination survey stages of the campaign, collected using the Mission Rabies App [15]. Additional geospatial datasets obtained from publicly available sources were also used for the regression analysis. Population density was obtained from WorldPop [16], as a raster file containing the number of people per hectare in 2011 with a 100 metre resolution. Poverty data with two different thresholds was also obtained from WorldPop [16], as two raster files containing the proportion of the people per grid square (1 km approximately) living on $1.25 and $2.00 a day, estimated in 2011. Land cover data was obtained from MASDAP [17] as a raster file with the topographical organization of Malawi in 2010 according to the Intergovernmental Panel on Climate Change (IPCC) 6-category scheme [18]. The R package *raster* [19] was used to manipulate and extract information from the raster datasets. All maps were plotted using the R package *ggmap* [20], using background tiles sourced from Stamen Design (which uses data from OpenStreetMap [21]) available under CC-BY 3.0 license.

### Data analysis

The R statistical software environment version 3.4.3 [22] was used for all data manipulation and analyses performed. Specific R packages used are described in the following sections.

### Estimation of vaccination coverage

During the survey stage, dog owners were asked how many dogs they owned and how many of those were vaccinated. The vaccination status of a dog was determined based on the owner’s verbal statement. Vaccination coverages were calculated based on the number of dogs reported as vaccinated, out of the total number of dogs surveyed. The 95% binomial confidence interval (CI) for coverage was calculated using the *binom*.*test* function from base R [22].

### Analysis of dog demographics

The dog population numbers were calculated using the Chapman estimator [23] for mark and recapture. The *ciChapman* function from the *recapr* package [24] was used to calculate the 95% CI for the dog population size, using the default bootstrap method. Due to the surveying method, these estimates only considered the owned population, which was expected to be extremely high based on previous reports. The total dog population was inferred through the proportion of owned dogs for each region. Dog:human ratios were calculated using the human population data from the 2018 Malawi Population and Census Preliminary Report [3]. The analysis on dog demographics regarding sex, age, confinement status, ownership status and neuter status was performed using data from the D2D stage as it was considered the most comprehensive dataset, including owned and stray populations. The Two-Sample t-test was used to identify any difference in the means of dog-owning households between rural and urban areas, using the *t*.*test* function from base R [22].

### Vaccination campaign logistics

A Two-Sample t-test was used to identify any difference in distance covered by the vaccination teams during the D2D stage between rural and urban areas, using the *t*.*test* function from base R [22].

### Regression analysis of vaccination coverage

The global positioning system (GPS) data obtained from the vaccination survey stage was used to create Convex Hull polygons containing the areas in which the different survey teams had worked each day. The polygons were attributed with averaged values of different variables inferred from the post-vaccination survey and geospatial datasets. This information was used to build logistic regression models that analysed the influence that several dog-related and geospatial factors have on the coverage achieved on each of these polygons.

### Identification and management of GPS outliers

Data from the vaccination coverage survey was analysed in order to detect any GPS outliers caused by the coordinate inaccuracy inherent of automated recording systems [25]. The inaccuracies were considered to happen at random, and needed to be identified and excluded before the assembly of the spatial polygons. The discrimination was performed by spatial clustering, using the *dbscan* function from the *fpc* package [26], in order to identify clusters of survey entries. Different values for the epsilon neighbourhood (eps) parameter were used depending on the district, due to their different distribution: 0.008 for urban Blantyre and urban Zomba, 0.013 for rural Blantyre and rural Zomba, and 0.015 for Chiradzulu. The minimum number of neighbours was set to 3 for all districts. Clusters containing less than 30% of the total points for that day and team were marked as outliers and ignored from the assembly of the polygons and the posterior regression analysis. From the 3669 vaccination survey entries, 60 were marked as outliers.

### Assembly of Convex Hull polygons

The GPS coordinates from the vaccination survey entries were organized according to day and team responsible, and turned into sets of Convex Hull polygons using the *gConvexHull* function from the *rgeos* package [27]. Overlapping polygons from entries surveyed on the same date were merged into a single polygon. Polygons composed by less than 6 surveyed households were considered to have insufficient sample size and therefore ignored, removing an extra 167 entries from the regression analysis. Information from the geospatial datasets was obtained for the area inside each of the polygons using the *extract* function from the *raster* package [19], and averaged to obtain single values for each polygon. Distance from the centre of the polygon to the closest city was calculated using the *nn2* function from the *RANN* package [28], using the coordinates of the Mission Rabies offices in Blantyre and Zomba as city centres. Variables related to the dog and human population were also averaged (for example, *Mean household occupation* or *Mean education level*) or used as proportions per polygon (for example, *Proportion of confined dogs* or *Proportion of female dogs*), depending on their nature. The variable *Mean education level* was created as a numerical scale representing the education level of the person surveyed, from 0 (no education) to 4 (higher education: undergraduate and postgraduate studies). The variable *Proportion of young dogs* represented the proportion of young (under 1 year of age) animals. The variable *Proportion of other animal ownership* was created as a proportion of the population owning chickens and/or pigs, in addition to dogs.

### Development of the logistic regression models

The purpose of the regression analysis was to obtain two logistic regression models, built using the *glm* function from base R [22], in order to determine the effect of different predictor variables on two different binomial responses. The response variable for the first model was whether adequate (over or equal to 70%) coverage was obtained in each polygon, supplied as a TRUE/FALSE response variable. This model will be referred to as the *Over the Threshold Model* (OTM). The second model investigated the effect of different predictor variables on absolute vaccination coverage, as the probability of a dog being vaccinated, supplied as a number of successes (dog vaccinated) and failures (unvaccinated dogs) in each polygon. This model will be referred to as the *Absolute Coverage Model* (ACM). The models were built using 139 entries, representing the 139 assembled polygons, which contain the aggregated attributes of 3442 entries after outlier detection and removing polygons with deficient number of households (S1 Dataset). The linearity of the numerical variables was tested using the Box-Tidwell Transformation test [29]. The transformation showed that linearity could not be assumed for some of the variables included in the ACM analysis. For this reason, these continuous variables were transformed into categorical. The variable “Proportion of female dogs” was turned into *Female majority*, being true when females composed over 50% of the dogs for that polygon. The variables *Proportion of confined dogs* and *Proportion of other animal ownership* were coded as levels high (at least 2/3), low (less than 1/3), and medium (in-between) based on the proportion of said category. The variable *Proportion of young dogs* was organized into three categories: [0–15] %, (15–30] %, and (30–45] %, since no polygon contained more than a 45% of young animals. The variables *Population density* and *Poverty (2*.*00 threshold)* were distributed accordingly into quartiles. These factorized variables were only used during the ACM analysis, substituting their continuous counterparts. To ease the interpretation of the influence of each variable on the vaccination coverage achieved (ACM), the estimated marginal means of the coverage achieved according to each of the predictive variables were calculated. This was performed using the *emmeans* package [30]. The effect of continuous variables (*Mean education level* and *Distance to the closest city*) were described using the quantile values. The variables considered for the regression analysis were: *Region*, *Setting*, *Mean household occupancy*, *Mean education level*, *Proportion of young dogs*, *Proportion of female dogs*, *Proportion of other animals*, *Ownership of other animals*, *Population density*, *Poverty* (both thresholds), *Land cover*, *Distance to the closest city*, and *Closest city*. Univariable logistic regression analyses were performed to test the statistical association of the individual variables with the outcome of interest (OTM or ACM). After this, multiple models were built for each of the responses separately, with the goal of selecting the OTM and ACM with the best fit. The models were built using combinations of the explanatory variables with univariable regression P values under 0.2 and meaningful interactions between them.

### Model selection

The performance of the models for the OTM analysis was studied using 5-fold cross validation [31], a process which divides the dataset into 5 sets of 5 subsets. In an alternated fashion, each 4 subsets of each set are used to build a model that is tested against the remaining subset. The process produces an area under the receiving operator curve (AUC), an averaged goodness of fit estimation from the 5 sequential analyses. The AUC for the models was estimated using the *ROCR* package [32]. The best model was selected comparing their respective AUC and their Akaike Information criterion (AIC) [33], looking for the highest AUC with the lowest AIC, in a compromise between predictive power and simplicity. The choice of the best model for the ACM analysis was based on the comparison of their AIC parameter alone, as the AUC can only be calculated for dichotomous (TRUE/FALSE) responses.

## Results

### Number of dogs vaccinated

A total of 89,032 dogs were vaccinated during the 2017 campaign. 55,526 dogs (62.4%) were vaccinated at the different SP set up across the working area, while 33,506 dogs (37.6%) were vaccinated during the D2D vaccination stage. From the 53,812 dogs found during D2D, 12,483 (23.2%) dogs were already vaccinated. From those already vaccinated, the great majority (88.4%) were immunized during the SP stage. Conversely, 1,661 (3.1%) dogs could not be vaccinated during the D2D stage, the most common reasons being problems handling the dog (62.8%) and owner consent (31.3%). The vaccination status for 6,162 (11.4%) dogs seen was not recorded. According to 11,421 owners asked during the D2D stage, the most common reasons for not attending a SP for vaccination were unawareness of the campaign (34.2%), unavailability (20.6%), distance (17.2%) and handling problems (16.2%). Responses indicating that participants considered the vaccine unnecessary or harmful represented a small percentage of the total answers (0.8%). According to household questionnaires carried out during the survey stage, the most common reasons for not attending a SP were handling problems (36.9%) and the dog being considered too young (27.2%), Table 2 includes a summary of the vaccination efforts during the SP and D2D stages in the different regions.

**Table 2:** Summary of vaccination numbers and estimated coverage per region. Coverage achieved with 95% CI also included.

Region	SP vaccinated	D2D vaccinated	Total vaccinated	Total Surveyed	Surveyed Vaccinated	Coverage (%)	Confidence Interval (95%)
**Blantyre Rural**	9149	6593	15742	587	486	82.8	(79.5–85.8)
**Blantyre Urban**	24782	9644	34426	1715	1466	85.5	(83.7–87.1)
**Chiradzulu Rural**	8822	5757	14579	753	626	83.1	(80.3–85.7)
**Zomba Rural**	8064	9948	18012	480	369	76.9	(72.8–80.6)
**Zomba Urban**	4709	1564	6273	134	113	84.3	(77–90)
**Total**	**55526**	**33506**	**89032**	**3669**	**3060**	**83.4**	**(82.2–84.6)**

### Estimation of vaccination coverage

**Fig 1:**
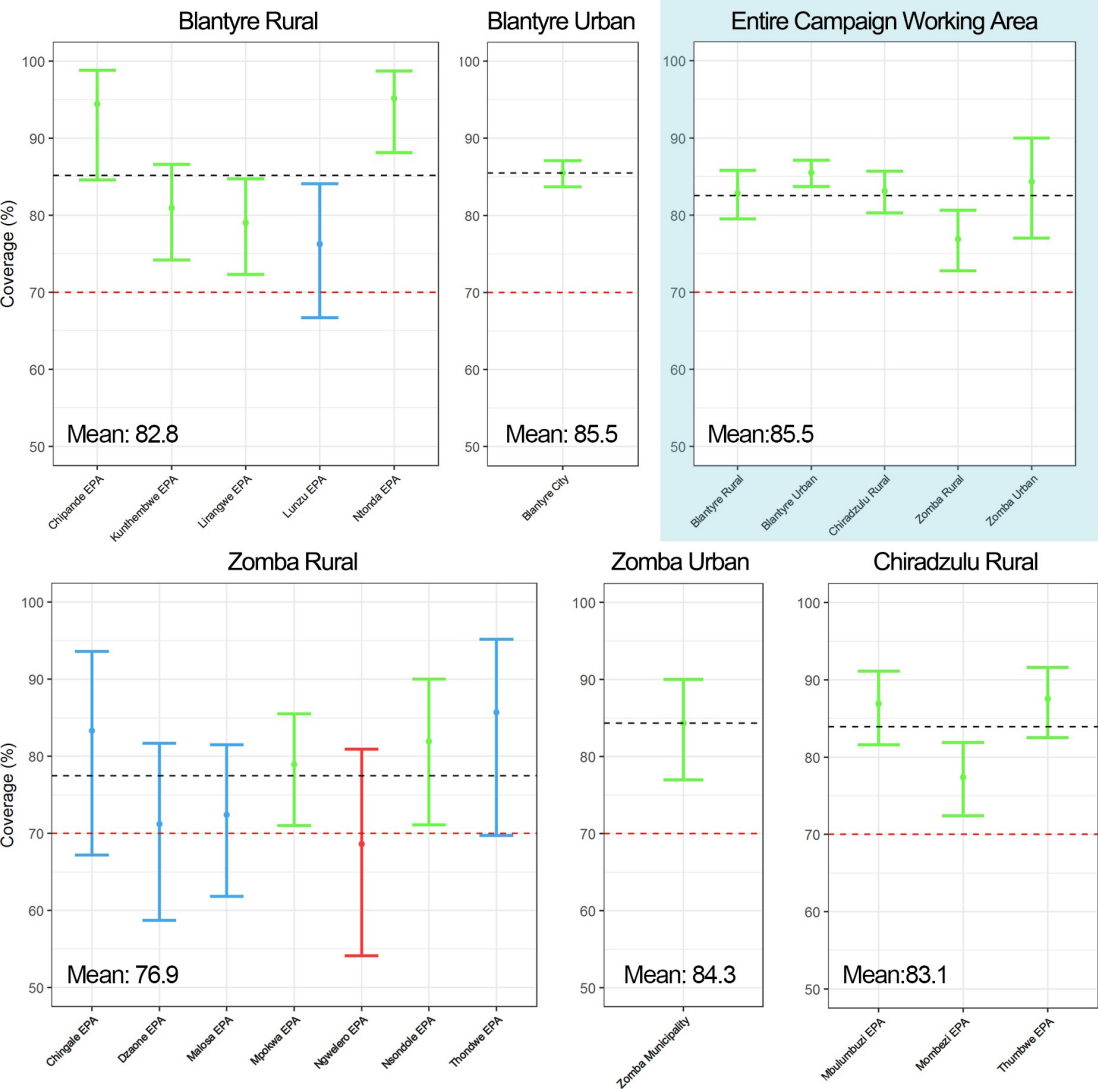
Plot of dog vaccination coverage per EPA. Coverages for the whole southern Malawi working area included. 95% confidence intervals presented by the vertical bars. EPAs whose coverage surpassed 70% are coloured in blue, while EPAS under 70% are coloured in red. EPAs whose coverage and lower 95% CI bound surpass 70% are coloured in green.

Out of the 3,669 dogs surveyed after the vaccination stages, 3,060 were identified as vaccinated. This identification was based on verbal confirmation by the owner, and supporting vaccination certificates were provided for 85.5% of the dogs reported as vaccinated The average vaccination coverage achieved across the working zone was estimated as 83.4% (95% CI: 82.2%– 84.6%). Separately, all 5 regions achieved vaccination coverages over 75% (Table 2), with coverages surpassing 70% in 16 out of the 17 EPAs that comprised the study area (Fig 1).

### Dog demographics

**Fig 2:**
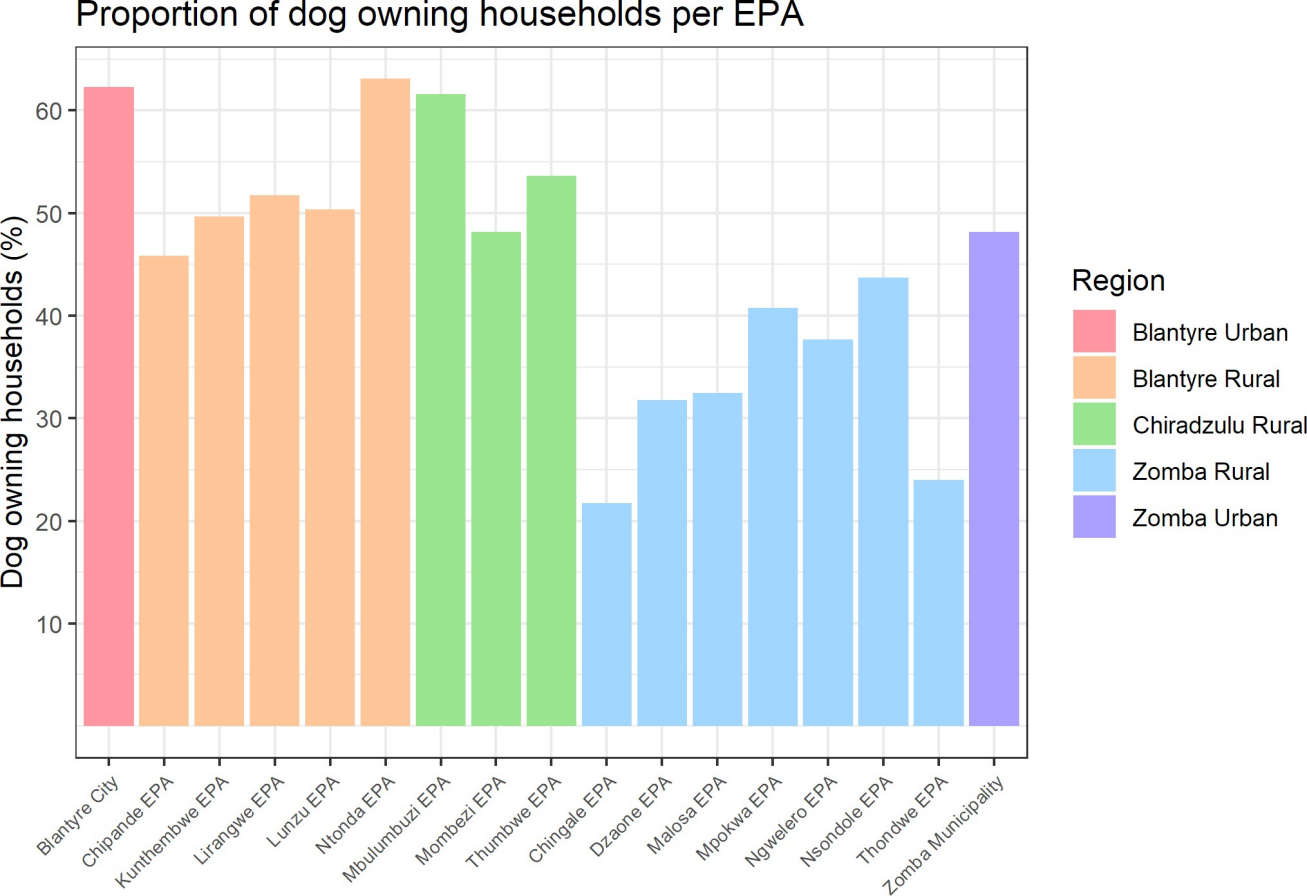
Bar plot of dog household ownership per EPA. EPAs coloured according to the region they belong to.

The dog population for the southern Malawi working zone was estimated as 107,574 dogs (95% CI: 106,049–109,179), with a dog:human ratio of 1:23. The great majority (99.23%) of the dogs found were owned. Table 3 summarizes dog ownership, dog population and dog:human ratios for the 5 districts. 52.6% of the households surveyed owned dogs. The mean number of dogs owned per surveyed household was 2 in urban Blantyre, 1.7 in rural Blantyre, Chiradzulu and rural Zomba, and 1.6 in urban Zomba. A graph representing the proportion of dog-owning households per EPA and district is shown in Fig 2. No statistically significant difference was found between the mean proportions of dog-owning households in urban and rural EPAs (P value: 0.35).

**Table 3:** Summary of ownership and dog population estimates per region. Estimates based on the D2D dataset.

Region	Owned dogs seen	Owned proportion	Dog-owning households	Dog population	Confidence Interval (95%)	Dog density (dog / km^2^)	Dog:Human ratio
**Blantyre Rural**	6959	99.91%	51.67%	19023	(18381–19752)	10.69	1:23.7
**Blantyre Urban**	20336	98.77%	62.27%	40770	(40014–41580)	171.31	1:19.6
**Chiradzulu Rural**	5755	99.77%	53.47%	17571	(17023–18173)	22.88	1:20.3
**Zomba Rural**	9975	99.6%	33.69%	23509	(22465–24692)	9.51	1:31.8
**Zomba Urban**	4258	98.72%	48.19%	7524	(7025–8152)	187.81	1:14
**Total**	**47283**	**99.23%**	**52.61%**	**107574**	**(106039–109179)**	**53.32**	**1:23**

During the D2D stage, a higher proportion (62.7%) of male dogs was found, and the majority (81.9%) of the dogs were adult (over one year of age). Only a minority (13.6%) of the dogs seen were neutered. According to household questionnaires, 82.7% of the dogs were kept for security and protection, while 12.4% were kept simply for companionship. The estimated proportion of dogs kept for security and companionship in urban Blantyre was 92.2% and 4%, respectively; 70.1% and 23% in rural Blantyre; 97% and 1.7% in rural Zomba; and 58.4% and 32.1% in Chiradzulu. All dogs surveyed in urban Zomba were reported to be kept for security purposes. 39.6% of the dogs were reported to be kept always under confinement, 34.6% were kept always unconfined, and 25.4% were left unconfined occasionally.

### Vaccination campaign logistics

An average of 32.8 dogs were vaccinated per hour/team during the SP stage, with 467.6 dogs vaccinated per day. During the D2D campaign, 14 dogs per hour/team were vaccinated on average, with 187.5 dogs vaccinated per day. Detailed vaccination per hour and day averages for each region can be found in Table 4. Regarding SP vaccinations, 50% of all the dogs vaccinated were vaccinated between 9:32 and 11:08 hours. 75% of all the dogs vaccinated were brought between 8:52 and 13:37 hours. The time distribution of dogs vaccinated during the SP stage is shown in S1 Fig. The teams responsible for the D2D vaccinations covered a mean of 19.2 km per day. The mean distance covered in urban areas was 20 km per day, while the mean distance covered in rural areas was 18.7 km per day. There was no significant difference in the mean distance covered per day between urban and rural settings (P value: 0.166).

**Table 4:** Summary of vaccination numbers per day and mean vaccinations per day/team and hour/team.

Region	Campaign	Mean vaccinated per day	Mean vaccinated per day / team	Mean vaccinated per hour / team
**Blantyre Rural**	**D2D**	68.7	37.2	10.6
	**SP**	113.2	58.8	30.9
**Blantyre Urban**	**D2D**	482.2	27.1	21.7
	**SP**	1261.2	75.9	38.1
**Chiradzulu Rural**	**D2D**	78.9	39.9	11.5
	**SP**	143.6	73.1	28.5
**Zomba Rural**	**D2D**	165.8	41.5	10.3
	**SP**	169.2	53.9	32.6
**Zomba Urban**	**D2D**	142.2	19.2	15.9
	**SP**	651	77.9	33.7
**Total averages**	**D2D**	**187.5**	**33**	**14**
	**SP**	**467.6**	**67.9**	**32.8**

### Convex Hull polygon determination

**Fig 3:**
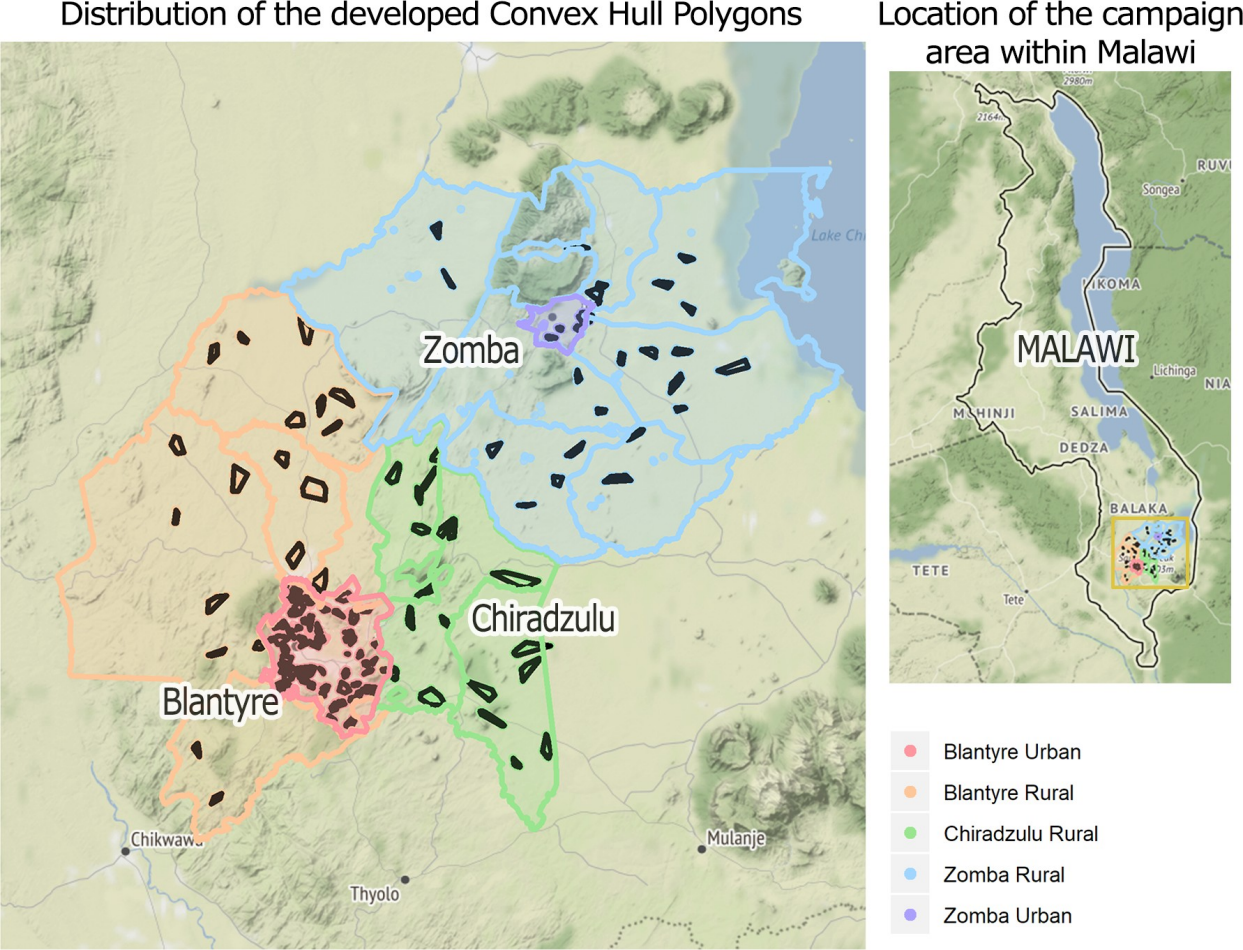
Topographical distribution of the 139 polygons used for the logistic regression. The position of the campaign working zone within Malawi is also shown. Background tiling by Stamen Design (maps.stamen.com, CC BY 3.0), with data by OpenStreetMap (ODbL).

The data from the survey stage of the campaign was organized spatially in 139 polygons, based on the areas covered by each team each day. Information from a total of 1,984 households and 3,442 distinct dogs was summarized into each polygon for the regression analysis, in addition to the associated geospatial data. Polygons were distributed as follows: 70 in urban Blantyre, 20 in rural Blantyre, 7 in urban Zomba, 23 in rural Zomba, and 20 in Chiradzulu. The spatial distribution of polygons across the working area is displayed in Fig 3. A descriptive summary by region of the attributes averaged through the Convex Hull polygons method is presented in Table 5.

**Table 5:** Descriptive summary by region of attributes averaged through the Convex Hull polygons in each region. The mean values for each variable are shown. “Other animal percentage” refers to the percentage of households owning other animals. “Education levels” refers to the education of the surveyed person, on a numerical scale from 0 (“no education”) to 4 (“under- or postgraduate studies”). “Population density” refers to the number of people per hectare (2011). “Poverty $2.00 proportion” refers to the proportion of people living on $2.00 a day (2011).

Variable	Blantyre Rural	Blantyre Urban	Chiradzulu Rural	Zomba Rural	Zomba Urban
**Houses per polygon**	14.4	13.9	22.2	8.9	13.0
**Dogs per household**	1.99	1.69	1.68	1.69	1.61
**Female dog proportion**	35.48%	30.89%	36.53%	29.27%	33.02%
**Young dog proportion**	13.68%	4.69%	11.31%	14.09%	0.68%
**Confined dog proportion**	13.47%	53.16%	31.85%	32.76%	21.34%
**Other animal proportion**	79.24%	35.83%	80.71%	74.42%	51.60%
**Household occupancy**	3.4	3.5	3.3	2.3	4.1
**Education level**	1.4	2.1	1.6	1.2	1.8
**Population density**	4.08	90.82	5.56	4.46	58.57
**Poverty $2.00 proportion**	77.16%	30.34%	76.37%	81.41%	58.58%

### Logistic regression analysis of adequate vaccination coverage (OTM)

Univariable logistic regression analyses were performed to identify predictive variables with a significant association with reaching adequate vaccination coverage. The results of this analysis are shown in S1 Table. *Region*, *Setting*, *Mean household occupancy*, *Mean education level*, *Proportion of young dogs*, *Proportion of female dogs*, *Ownership of other animals*, *Poverty level* (both thresholds), *Distance to the closest city* and *Closest city*, having P values under 0.2, were the variables considered for their inclusion in the multivariable models. The model chosen after the 5-fold cross validation was composed by *Mean education level* and *Proportion of young animals* as the only predictor variables. Adequate coverage was found to be positively associated with higher education levels in the area, with an odds ratio of 2.933 (95% CI: 0.957–8.924, P value: 0.06). Higher proportions of young animals were associated with lower odds of reaching adequate coverage, with an odds ratio of 0.951 (95% CI: 0.905–0.997, P value 0.044). The model had a moderate predictive power (AUC = 0.724).

### Logistic regression analysis of absolute vaccination coverage (ACM)

**Fig 4:**
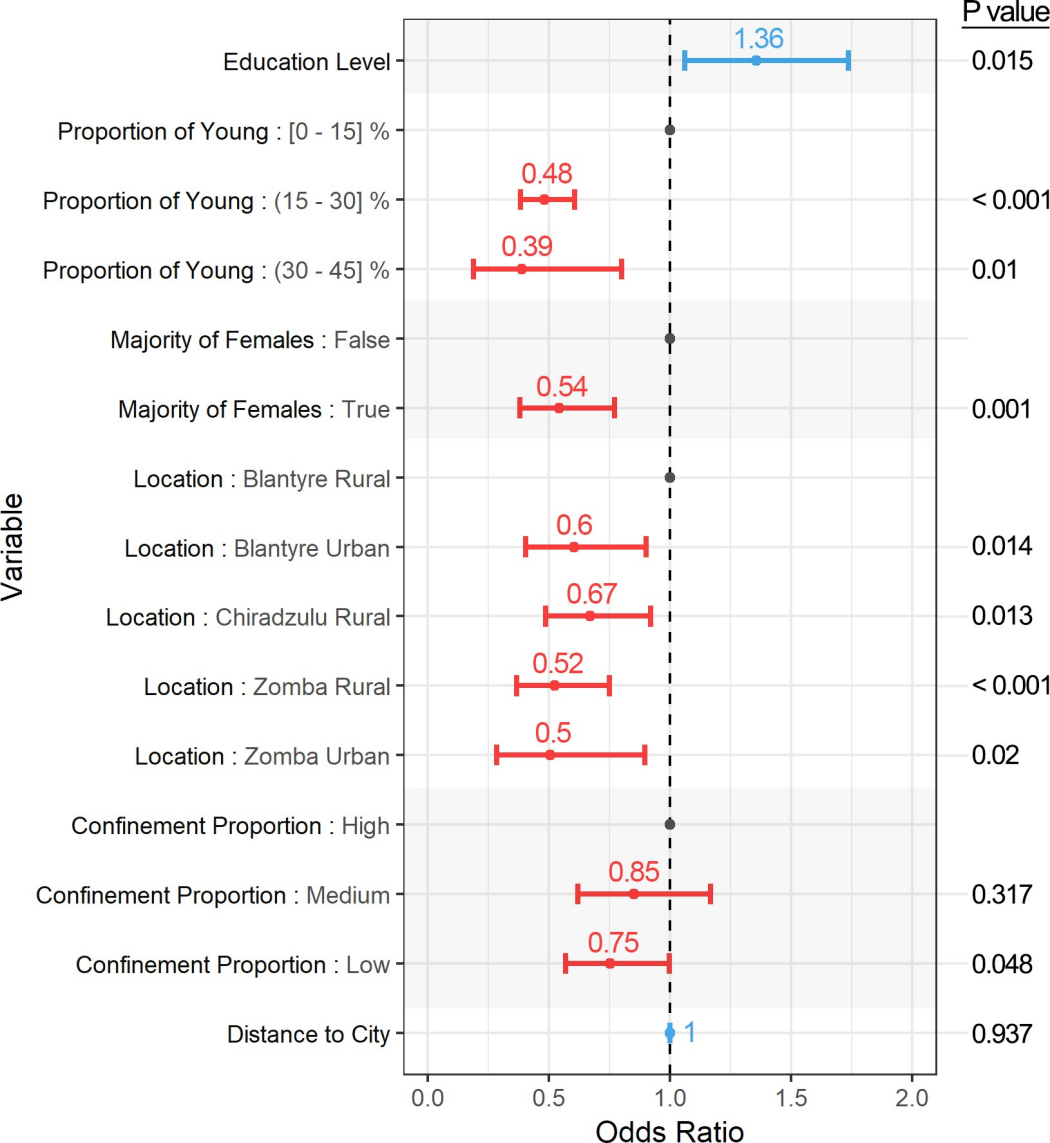
Graphical representation of the chosen regression model predicting absolute coverage (ACM). The 95% CI is represented as horizontal bars. The value for the odds ratio is indicated above the 95% CI. A positive relationship between the variable and the response is coloured in red (odds ratio > 1.000), while a negative relationship is coloured in blue (odds ratio < 1.000). Baseline categories are coloured in grey. P values are shown on the left.

**Fig 5:**
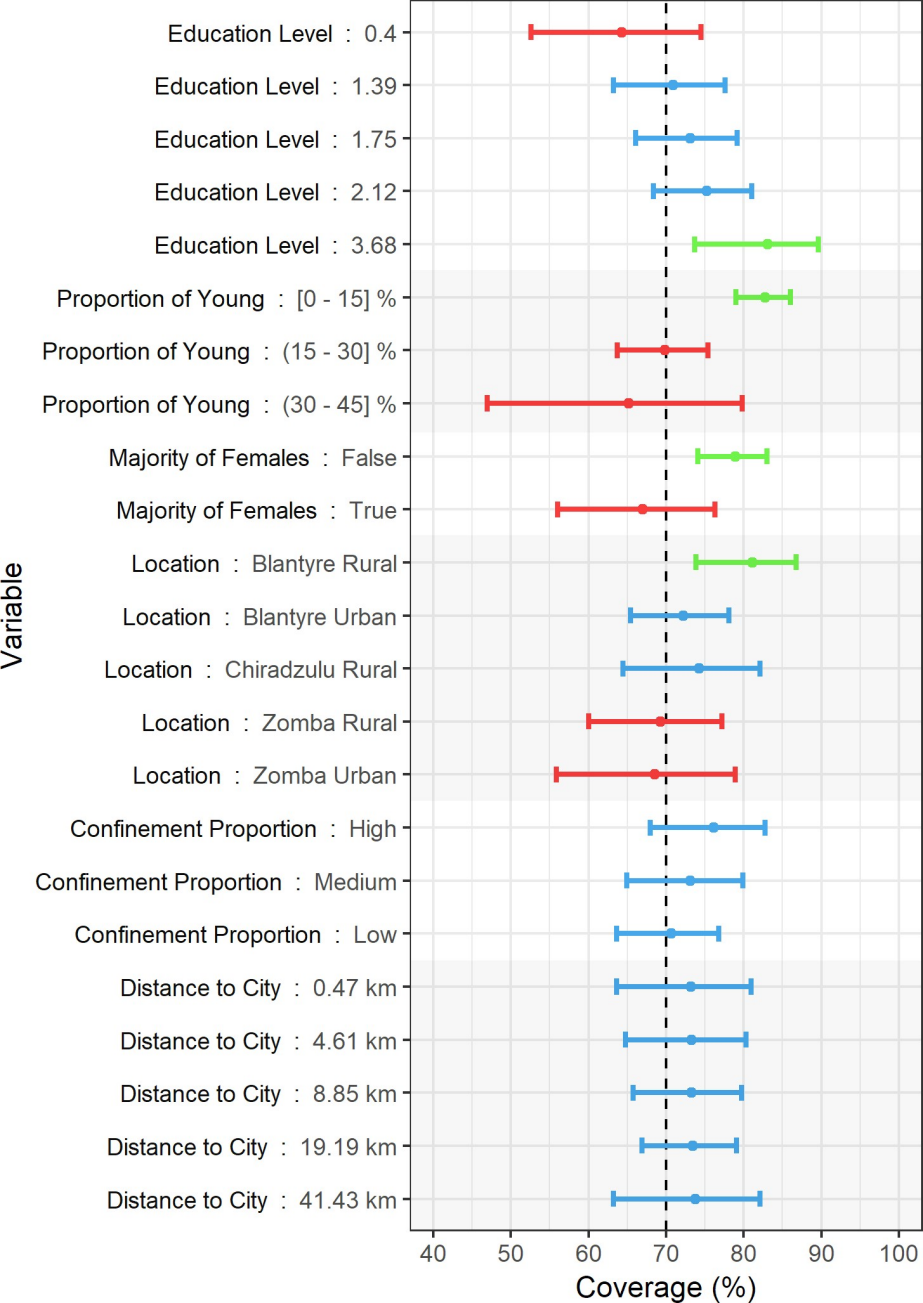
Graphical representation of the estimated marginal means analysis from the absolute coverage model (ACM). The vertical bar represents the 70% vaccination coverage threshold. The 95% CI is represented as horizontal bars. Estimated coverages under 70% are coloured in red. Estimated coverages over 70% are coloured in blue. Estimated coverages whose lower 95% CI bound surpass 70% are coloured in green.

The results of the univariable regression analysis for absolute vaccination coverage as response variable are shown in S2 Table. *Region*, *Setting*, *Mean household occupancy*, *Mean education level*, *Proportion of young dogs*, *Majority of female dogs*, *Proportion of confined dogs*, *Proportion of households owning other animals*, *Population density*, *Poverty* (both thresholds), *Land cover* and *Distance to the closest city* were the variables with P values under 0.2 and therefore considered for their inclusion in the models. The final model was chosen based on the AIC and its simplicity. It showed that absolute coverage (represented by vaccination probability) was positively associated with the mean education level and high proportions (over 2/3) of confined dogs, as polygons with low and medium confinement showed a decrease in the odds ratio. Polygons with an increased proportion of young animals or a majority of female dogs had decreased odds ratios. The distance from the centre of the polygon to the nearest city was found to have a non-significant effect, with an odds ratio of 1.000. In addition, Blantyre rural was associated with increased odds ratios compared to the rest of districts. This analysis is represented in Fig 4, showing the odds ratio and CI for each of the predictive variables. The results from the estimated marginal means analysis are represented numerically in S3 Table, and graphically in Fig 5, showing the same behaviour for vaccination coverage as described above. The mean coverage increased with education level and the proportion of confined dogs, and decreased with a majority of females and with an increasing proportion of young dogs. A variation in mean coverage was observed according to the location, while distance to the closest city appeared to produce a negligible effect.

## Discussion

This study demonstrates the feasibility of vaccinating a large number of dogs at high coverage in a low-resource setting through a range of geographical areas in southern Malawi. The campaign vaccinated more than 89,000 dogs across rural and urban settings, reaching an average coverage of 83.4% with the three individual districts achieving vaccination coverages over the 70% minimum recommended by the WHO [5]. The timespan between the vaccination stages and the post-vaccination survey, caused by the wide scale of the project, made the identification of vaccinated stray dogs unfeasible. However, although these estimates study only the owned population, they are considered accurate due to the very low percentage of stray dogs in the population (less than 0.3% according to this study).

The vaccination coverage achieved is the highest ever reported in southern Malawi [11, 14]. Very few canine rabies vaccination campaigns in SSA have reached such large numbers of dogs vaccinated and high coverage [4, 8, 9], like the 80.3% reached in an agro-pastoralist region of Tanzania [34]. Our report continues to demonstrate that dogs in rural areas of SSA can indeed be efficiently vaccinated reaching high vaccination coverages. It is important to note that Mission Rabies had worked in Malawi for two years before this campaign, which might have improved the results through increased disease and campaign awareness, and acceptance of the procedure.

Despite the fact that dog communities in SSA are mostly owned [9], D2D stages complementing the SP vaccination are often essential in order to reach a wide proportion of this populations in settings where attendance to SP clinics is suboptimal [11, 34]. Despite vaccinating over 55,500 dogs during the SP stage, only 23% of the dogs found in the following D2D vaccination stage were identified as already vaccinated. The most common reasons reported for not attending a SP (unawareness, unavailability, problems handling the dog, and distance) were also reported as the most common in other campaigns based on different settings [12, 35–38], highlighting some of the main aspects to improve in subsequent vaccination campaigns.

The dog:human ratio estimated for the urban Blantyre area (1:19.6) is very similar to the 1:18.1 ratio estimated during the 2015 campaign [11]. The ratios estimated show a higher dog proportion in urban areas, and a significantly lower dog proportion in rural areas, when compared with the average ratios for African urban and rural areas estimated by Knobel *et al*. [39]. The presence of a majority of owned dogs (99.2% in this study) is considered a fundamental characteristic of African dog populations [9], with most dogs (82.7% in this study) being kept for security or protection purposes [40–43]. The proportion of dog-owning households in Blantyre city (62,3%) is similar to that reported during the 2015 campaign [11], with the dog population also being composed of nearly 80% adult dogs. Other studies have also reported an overrepresentation of male dogs in SSA domestic populations [9].

The use of regression modelling to determine and estimate the influence of dog and geospatial factors on different aspects of vaccination campaigns is a novel approach able to produce insights on their performance [12, 44]. The model studying factors associated with adequate coverage (OTM) only included two predictors: mean education level and proportion of young dogs. An increase of 1 in the education level nearly triples the odds of achieving adequate coverage, while an increase of 1% in the proportion of young (less than 1 year of age) animals produces a slight decrease in the odds of adequate coverage. The identification of only two significant variables for the OTM analysis could be explained by the overall success of the campaign, with coverages under 70% occurring in only 20 out of the 139 polygons. This is further supported by the fact that none of the factors related to location (*Region*, *Setting* or variables containing geospatial data) were significantly associated with achieving adequate coverage.

The model studying the variability in coverage (ACM) incorporated a wider set of predictive factors. In this case, and as represented in Fig 5, higher education levels and higher proportions of confined animals were positively associated with higher vaccination coverages. Conversely, increasing proportions of young dogs and populations consisting of a majority of females were associated with lower vaccination coverages. Dogs in all regions showed decreased odds of vaccination compared to rural Blantyre, an effect likely caused by having reached very high coverages (around 95%) in the Chipande and Ntonda EPAs, as shown in Fig 1. The distance to the closest city appeared have a negligible effect on vaccination coverage variation.

Improvement in rabies awareness through the development of education campaigns has been recognised as a key factor in order to control the infection [5]. The delivery of these activities has been proven beneficial not only to provide the community with accurate information on disease prevention, but to improve their attitude concerning dog vaccination and engagement with rabies surveillance [13, 45, 46]. A similar regression analysis also showed that education was positively associated with the owner’s intention to vaccinate their dog [44]. For similar reasons, it is logical that dogs from areas with higher education levels show increased odds of rabies vaccination. Female dogs have been reported to be less likely to be brought to SP compared to male dogs [12], which agrees with this study showing areas with a female majority having halved odds. This behaviour might be explained by male dogs being considered more valuable, as they are more often used for security [9]. In a similar manner, areas with proportions of young dogs higher than 15% are also associated with lower vaccination coverages. The dog being considered too young to vaccinate was the second most common reason given by the owners during the post-vaccination survey stage when asked about their failure to attend a SP. This misconception has also been reported previously [11, 12, 34], making young dogs a difficult demographic to reach through SP vaccination campaigns. It is necessary for this unfounded belief to be addressed via educational campaigns, since many young animals still remain at risk for months, even while the vaccine has been proven to be both safe and effective in puppies [47]. The deterrent effect of distance on attendance to healthcare facilities has been previously reported [48, 49], in addition to the specific effect of distance on attendance to static vaccination clinics [12, 44]. However, in this study, the effect of distance on absolute coverage was found to be insignificant. The inclusion of more diverse GIS data, unavailable at the time of this study (for example, land use or housing type), might improve the fitness of further models and help explain the behaviour of variables such as *Location* and *Distance to closest city*. In the same manner, the unavailability of more recent (and thus more representative) GIS datasets regarding population density and poverty levels might have caused their exclusion as significant variables in the models.

Using strategies similar to those used during the 2015 campaign [12] and adapting them to rural settings, the 2017 campaign managed to reach a higher coverage working over a wider area during a larger timespan. Thanks to an increased vaccination focus in these commonly considered “hard to reach” rural areas, vaccination coverages of over 75% were achieved in rural Blantyre, rural Zomba and Chiradzulu, with very similar coverages as those achieved in the urban regions. This study shows the feasibility of delivering high vaccination coverage homogeneously across a wide range of settings within three districts in southern Malawi, an approach that may be used as a template for future dog vaccination programmes in other areas of SSA.

## Conclusion

This is the first reported large-scale dog vaccination campaign in SSA covering multiple districts within both urban and rural settings. Over 89,000 dogs were vaccinated at coverages over 70% throughout Blantyre, Zomba and Chiradzulu districts. The regression analysis determined factors influencing vaccination coverage and showed that, for this approach, the intrinsic geographical differences between urban and rural settings had no effect on achieving at least 70% coverage. The protocols described in this study were remarkably successful in southern Malawi and could be adapted to achieve high coverage and high vaccination numbers in similar areas of SSA spanning urban and rural populations.

## Supporting information

S1 Dataset Survey polygons dataset. Csv file containing the attributes of the 139 polygons used in the regression analysis. Due to privacy reasons, GPS locations were removed from the dataset.
(CSV)

S1 Table Univariable logistic regression analysis results testing association of variables with adequate coverage (OTM). Analysis performed using 139 polygons, containing the aggregated attributes of 3442 data entries.
(DOCX)

S2 Table Univariable logistic regression analysis results testing association of variables with absolute coverage (ACM). Analysis performed using 139 polygons, containing the aggregated attributes of 3442 data entries.
(DOCX)

S3 Table Estimated marginal means analysis results of the absolute coverage model (ACM). Analysis performed using 139 polygons, containing the aggregated attributes of 3442 data entries.
(DOCX)

S1 Fig Histogram of dog vaccination times at SPs. Bars are coloured according to the region the SPs were set up in.
(TIFF)
