# Supplementary material for: Implementation of a mass canine rabies vaccination campaign in both rural and urban regions in southern Malawi
Source: PLoS Negl Trop Dis. 2020 Jan 23;14(1):e0008004. doi: 10.1371/journal.pntd.0008004 (PMC6999910; doi:10.1371/journal.pntd.0008004)
Supplement: S1 Table — Analysis performed using 139 polygons, containing the aggregated attributes of 3442 data entries. (DOCX) [file pntd.0008004.s002.docx]

| **Variable** | **Odds Ratio** | **Confidence Interval (95%)** | **Standard Error** | **P Value**  **(α = 0.05)** |
| --- | --- | --- | --- | --- |
| **REGION** |  | | | |
| Blantyre Rural | 1 | Baseline Category | | |
| Blantyre Urban | 1.37 | ( 0.33 – 5.72 ) | 0.73 | 0.67 |
| Chiradzulu Rural | 1.59 | ( 0.24 – 10.7 ) | 0.97 | 0.63 |
| Zomba Rural | 0.33 | ( 0.07 – 1.48 ) | 0.76 | 0.15 |
| Zomba Urban | > 999 | ( <0.001 - >999) | 1495.3 | 0.99 |
| **SETTING** |  | | | |
| Rural | 1 | Baseline Category | | |
| Urban | 2.24 | ( 0.86 – 5.82 ) | 0.49 | 0.1 |
| **MEAN HOUSEHOLD OCCUPANCY** |  | | | |
|  | 1.61 | ( 0.83 – 3.14 ) | 0.34 | 0.16 |
| **MEAN EDUCATION LEVEL** |  | | | |
|  | 4.46 | ( 1.55 – 12.82 ) | 0.54 | < 0.01 |
| **PROPORTION OF YOUNG DOGS** | 1 | Baseline Category | | |
|  | 0.94 | ( 0.89 – 0.98 ) | 0.02 | < 0.01 |
| **PROPORTION OF FEMALE DOGS** |  | | | |
|  | 12.61 | ( 0.32 – 504.52 ) | 1.88 | 0.18 |
| **PROPORTION OF CONFINED DOGS** |  | | | |
|  | 2.26 | ( 0.53 – 9.66 ) | 0.74 | 0.27 |
| **OWNERSHIP OF OTHER ANIMALS** |  | | | |
|  | 0.2 | ( 0.04 – 1.18 ) | 0.89 | 0.08 |
| **POPULATION DENSITY** |  | | | |
|  | 1 | (1 – 1.01) | < 0.01 | 0.65 |
| **POVERTY (1.25)** |  | | | |
|  | 0.11 | ( 0.02 – 0.81 ) | 1 | 0.03 |
| **POVERTY (2.00)** |  | | | |
|  | 0.13 | ( 0.02 – 0.93 ) | 0.99 | 0.04 |
| **LAND COVER** |  | | | |
| LC1 | 1 | Baseline Category | | |
| LC2 | 0.22 | ( 0.01 – 3.98 ) | 1.47 | 0.31 |
| LC3 | 1.38 | ( 0.13 – 14.41 ) | 1.2 | 0.79 |
| LC5 | 5 | ( 0.42 – 59.66 ) | 1.26 | 0.2 |
| **DISTANCE TO THE CLOSEST CITY** |  | | | |
|  | 1 | ( 1 – 1 ) | 0 | 0.18 |
| **CLOSEST CITY** |  | | | |
| Blantyre | 1 | Baseline Category | | |
| Zomba | 0.48 | (0.18 – 1.27 ) | 0.5 | 0.14 |
