# Supplementary material for: Implementation of a mass canine rabies vaccination campaign in both rural and urban regions in southern Malawi
Source: PLoS Negl Trop Dis. 2020 Jan 23;14(1):e0008004. doi: 10.1371/journal.pntd.0008004 (PMC6999910; doi:10.1371/journal.pntd.0008004)
Supplement: S2 Table — Analysis performed using 139 polygons, containing the aggregated attributes of 3442 data entries. (DOCX) [file pntd.0008004.s003.docx]

| **Variable** | **Odds Ratio** | **Confidence Interval (95%)** | **Standard Error** | **P Value**  **(α = 0.05)** |
| --- | --- | --- | --- | --- |
| **REGION** |  | | | |
| Blantyre Rural | 1 | Baseline Category | | |
| Blantyre Urban | 1.2 | ( 0.93 – 1.56 ) | 0.13 | 0.17 |
| Chiradzulu Rural | 0.98 | ( 0.74 – 1.32 ) | 0.15 | 0.91 |
| Zomba Rural | 0.56 | ( 0.41 – 0.78 ) | 0.16 | < 0.001 |
| Zomba Urban | 0.95 | ( 0.59 - 1.52 ) | 0.24 | 0.82 |
| **SETTING** |  | | | |
| Rural | 1 | Baseline Category | | |
| Urban | 1.36 | ( 1.14 – 1.63 ) | 0.09 | < 0.01 |
| **MEAN HOUSEHOLD OCCUPANCY** |  | | | |
|  | 1.26 | ( 1.11 – 1.45 ) | 0.07 | < 0.01 |
| **MEAN EDUCATION LEVEL** |  | | | |
|  | 1.66 | ( 1.39 – 1.99 ) | 0.09 | < 0.001 |
| **PROPORTION OF YOUNG DOGS** |  |  |  |  |
| [0 – 15] % | 1 | Baseline Category | | |
| (15 – 30] % | 0.46 | ( 0.38 – 0.56 ) | 0.1 | < 0.001 |
| (30 – 45] % | 0.47 | ( 0.23 – 0.94 ) | 0.36 | 0.03 |
| **MAJORITY OF FEMALE DOGS** |  | | | |
| False | 1 | Baseline Category | | |
| True | 0.5 | ( 0.37 – 0.68 ) | 0.15 | <0.001 |
| **PROPORTION OF CONFINED DOGS** |  | | | |
| High | 1 | Baseline Category | | |
| Medium | 0.78 | ( 0.58 – 1.05 ) | 0.15 | 0.1 |
| Low | 0.64 | ( 0.51 – 0.8 ) | 0.12 | < 0.001 |
| **OWNERSHIP OF OTHER ANIMALS** |  | | | |
| High | 1 | Baseline Category | | |
| Medium | 1.25 | ( 1.01 – 1.54 ) | 0.11 | 0.04 |
| Low | 1.78 | ( 1.39 – 2.27 ) | 0.13 | < 0.001 |
| **POPULATION DENSITY** |  | | | |
| [0 , 4.35] | 1 | Baseline Category | | |
| (4.35, 8.17] | 1.1 | ( 0.86 – 1.4 ) | 0.12 | 0.44 |
| (8.17, 54.5] | 1.82 | ( 1.42 – 2.33 ) | 0.13 | <0.001 |
| (54.5, 489] | 1.47 | ( 1.13 – 1.92 ) | 0.13 | < 0.01 |
| **POVERTY (1.25)** |  | | | |
|  | 0.36 | ( 0.25 – 0.52 ) | 0.19 | < 0.001 |
| **POVERTY (2.00)** |  | | | |
| [0, 0.248] | 1 | Baseline Category | | |
| (0.248,0.546] | 1 | ( 0.75 – 1.33 ) | 0.14 | 0.99 |
| (0.546,0.801] | 0.71 | ( 0.55 – 0.93 ) | 0.13 | 0.01 |
| (0.801,0.882] | 0.55 | ( 0.43 – 0.72 ) | 0.14 | < 0.001 |
| **LAND COVER** |  | | | |
| LC1 | 1 | Baseline Category | | |
| LC2 | 1.13 | ( 0.59 – 2.15 ) | 0.33 | 0.71 |
| LC3 | 1.41 | ( 0.92 – 2.16 ) | 0.22 | 0.11 |
| LC5 | 2.21 | ( 1.44 – 3.41 ) | 0.22 | < 0.001 |
| **DISTANCE TO THE CLOSEST CITY** |  | | | |
|  | 1 | ( 1 – 1 ) | 0 | < 0.001 |
| **CLOSEST CITY** |  | | | |
| Blantyre | 1 | Baseline Category | | |
| Zomba | 0.68 | ( 0.55 – 0.84 ) | 0.11 | < 0.001 |
