# Supplementary material for: Implementation of a mass canine rabies vaccination campaign in both rural and urban regions in southern Malawi
Source: PLoS Negl Trop Dis. 2020 Jan 23;14(1):e0008004. doi: 10.1371/journal.pntd.0008004 (PMC6999910; doi:10.1371/journal.pntd.0008004)
Supplement: S3 Table — Analysis performed using 139 polygons, containing the aggregated attributes of 3442 data entries. (DOCX) [file pntd.0008004.s004.docx]

| **Variable** | **Category** | **Estimated Coverage** | **Confidence Interval (95%)** | **Standard Error** |
| --- | --- | --- | --- | --- |
| **Mean Education** | 0.4 | 64.3 | ( 52.59 - 74.52 ) | 0.06 |
|  | 1.39 | 70.92 | ( 63.2 - 77.59 ) | 0.04 |
|  | 1.75 | 73.1 | ( 66.05 - 79.14 ) | 0.03 |
|  | 2.12 | 75.27 | ( 68.4 - 81.06 ) | 0.03 |
|  | 6.68 | 83.08 | ( 73.64 - 89.62 ) | 0.04 |
| **Proportion of Young** | [0 – 15] % | 82.78 | ( 79.01 - 85.99 ) | 0.02 |
|  | (15 – 30] % | 69.86 | ( 63.71 - 75.37 ) | 0.03 |
|  | (30 – 45] % | 65.17 | ( 46.95 - 79.82 ) | 0.09 |
| **Majority of Females** | False | 78.9 | ( 74.12 - 83 ) | 0.02 |
|  | True | 66.94 | ( 56.04 - 76.28 ) | 0.05 |
| **Location** | Blantyre Rural | 81.14 | ( 73.88 - 86.74 ) | 0.03 |
|  | Blantyre Urban | 72.22 | ( 65.43 - 78.12 ) | 0.03 |
|  | Chiradzulu Rural | 74.22 | ( 64.41 - 82.07 ) | 0.05 |
|  | Zomba Rural | 69.29 | ( 60.01 - 77.21 ) | 0.04 |
|  | Zomba Urban | 68.5 | ( 55.84 - 78.9 ) | 0.06 |
| **Confinement Proportion** | High | 76.15 | ( 67.93 - 82.79 ) | 0.04 |
|  | Medium | 73.09 | ( 67.93 - 82.79 ) | 0.04 |
|  | Low | 70.62 | ( 63.61 - 76.78 ) | 0.03 |
| **Distance to City** | 0.47 km | 73.19 | ( 63.63 - 80.99 ) | 0.04 |
|  | 4.61 km | 73.24 | ( 64.77 - 80.3 ) | 0.04 |
|  | 8.85 km | 73.3 | ( 65.74 - 79.71 ) | 0.04 |
|  | 19.19 km | 73.44 | ( 66.91 - 79.09 ) | 0.03 |
|  | 41.43 km | 73.74 | ( 63.18 - 82.13 ) | 0.05 |
