# Supplementary figures and images for: Implementation of a mass canine rabies vaccination campaign in both rural and urban regions in southern Malawi
Source: PLoS Negl Trop Dis. 2020 Jan 23;14(1):e0008004. doi: 10.1371/journal.pntd.0008004 (PMC6999910; doi:10.1371/journal.pntd.0008004)

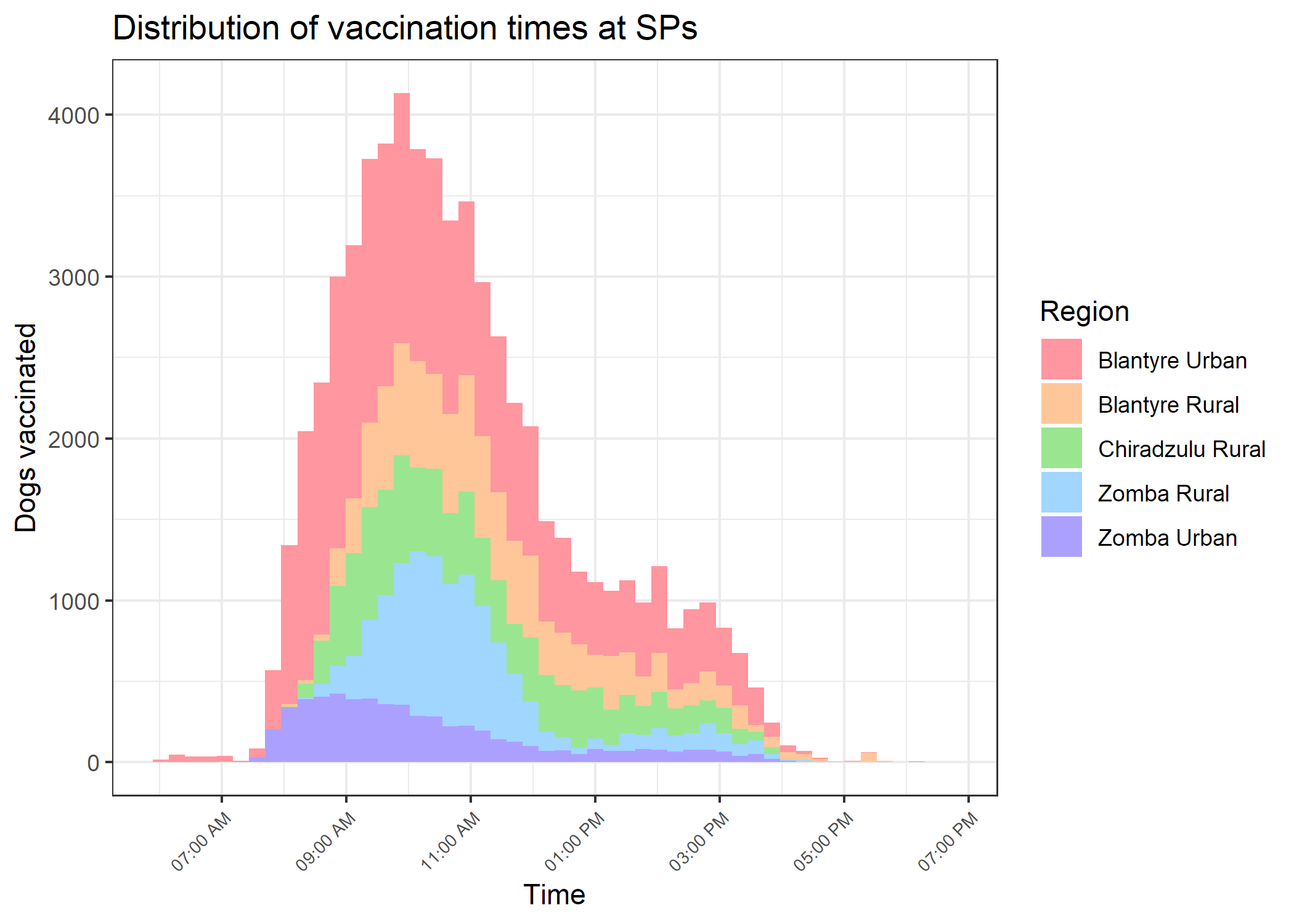

Supplement: S1 Fig — Bars are coloured according to the region the SPs were set up in. (TIFF) [file pntd.0008004.s005.tiff]
